# Lightweight Gait-Based Person Identification via Transmit-Channel Differential Encoding with MIMO Millimeter-Wave Radar

**DOI:** 10.3390/s26196056

**Published:** 2026-09-24

**Authors:** Hongming Shen, Chenxiao Zhang, Zhiming Sun, Nanfeng Mi, Lei Fang, Mei Ge, Xia Wang

**Affiliations:** School of Microelectronics and School of Integrated Circuits, Nantong University, Nantong 226019, China; hmshen@ntu.edu.cn (H.S.); chenxiao_zhang@stmail.ntu.edu.cn (C.Z.); zhimingsun@stmail.ntu.edu.cn (Z.S.); guifeng@stmail.ntu.edu.cn (N.M.); 2636320007@stmail.ntu.edu.cn (L.F.); meige@ntu.edu.cn (M.G.)

**Keywords:** mmWave radar, micro-Doppler, gait biometrics, multi-transmit-channel differential encoding, Asym-CRNN

## Abstract

Millimeter-wave (mmWave) radar enables non-contact human identification by exploiting gait micro-Doppler signatures without capturing visual imagery. Nevertheless, practical deployment encounters several critical challenges: strong torso echoes that obscure faint limb-motion features, channel-dependent responses caused by unconstrained walking styles, and computational limitations on edge hardware. This paper proposes a lightweight gait-based human identification method for multiple-input multiple-output (MIMO) radar systems, which integrates adaptive Gamma correction, multi-transmit-channel differential encoding, and an asymmetric-pooling convolutional recurrent neural network (Asym-CRNN). Specifically, the differences across the three transmit-channel responses of the three-transmit four-receive (3T4R) radar are encoded into a three-channel spatiotemporal representation, and the compact network retains temporal resolution to facilitate efficient gait discrimination. Evaluated on a self-collected five-subject dataset with acquisition-sequence-level train-test separation, the proposed method achieves closed-set accuracies of 89.62% and 99.52% under 1 s and 6 s observation windows, respectively. For open-set scenarios, the method attains a registered-versus-unregistered area under the receiver operating characteristic curve (AUROC) of 0.9869. With a maximum softmax probability (MSP) threshold of 0.85, the method rejects 91.88% of unregistered human samples and all tested pet-interference samples. Furthermore, the model achieves an accuracy of 86.98% using only single-channel IDRad inputs. The exported model requires merely 4.32 MB of storage. Deployed on a Raspberry Pi 5 platform, the acquisition-processing pipeline runs using only two CPU cores with CPU-only execution: one core continuously receives and buffers raw radar data, whereas the other processes previously acquired observation windows. The per-window processing latency reaches 715.7 ms for the 1 s window and 4366.2 ms for the 6 s window, including network inference times of 6.7 ms and 15.2 ms, respectively. The non-causal bidirectional long short-term memory (Bi-LSTM) generates one identity decision once each observation window is fully collected. These results verify the feasibility of the proposed method for privacy-preserving indoor monitoring at small scales on resource-constrained edge devices.

## 1. Introduction

With the growth of smart indoor environments, non-contact human sensing has become important for privacy-sensitive monitoring and interaction. It has potential applications in privacy-critical and security-sensitive scenarios, including home health monitoring, personalized human-machine interaction, and unobtrusive identity authentication [1,2]. Gait is a distinctive behavioral biometric that provides long-distance, non-cooperative, and spoof-resistant identity cues. Compared with static physiological traits such as fingerprints, irises, and faces, gait reflects individual motion patterns, including limb-swing amplitude, step rhythm, and torso coordination. It can provide continuous and implicit temporal discriminative cues for reliable identity verification [3,4,5].

Traditional gait sensing solutions predominantly rely on optical cameras or wearable inertial sensors. Vision-based methods capture explicit visual features, such as faces, body shapes, and clothing, which raises privacy concerns in private indoor environments. Moreover, vision-based sensing is susceptible to illumination changes and occlusion. Wearable devices also have inherent limitations, including discomfort, low user compliance, and limited battery life, making them incapable of supporting long-term unobtrusive monitoring. As an alternative, millimeter-wave (mmWave) radar enables human sensing by transmitting and receiving radio-frequency echoes without capturing optical images. It offers privacy preservation, robustness to lighting conditions, and limited penetration through certain non-metallic obstacles [1,3].

Frequency-modulated continuous-wave (FMCW) radar can extract micro-Doppler signatures for human motion sensing. During walking, motions of the torso, limbs, and other body parts produce time-varying frequency modulation in radar echoes [3]. The micro-Doppler time-frequency spectrogram characterizes the instantaneous radial-velocity distribution of different body parts as a two-dimensional time-frequency map. These time-frequency patterns contain person-specific gait information. Therefore, micro-Doppler spectrograms have been widely adopted for radar-based human identification and gait authentication [4,6,7,8]. Micro-Doppler spectrograms and radar point clouds provide complementary representations of human motion [3,9]. Micro-Doppler spectrograms emphasize continuous time-velocity patterns associated with limb micromotions, whereas radar point clouds retain explicit spatial and geometric information [3,10,11]. Spectrogram-based methods can avoid some target-detection and point-cloud-clustering operations, but the computational cost of either representation depends on the specific preprocessing pipeline and network architecture.

Radar micro-Doppler-based gait recognition has progressed substantially over the past decade. Existing studies have shown that micro-Doppler modulations induced by articulated motion provide discriminative features for human identification [3]. Early research on Ultra-Wideband (UWB) radar-based human identification further demonstrated that micro-Doppler signals contain discriminative information applicable for individual differentiation [12,13]. In recent years, deep learning algorithms have become the dominant technical paradigm in this field. Numerous studies have integrated deep convolutional or temporal convolutional networks with spectrogram analysis for low-power FMCW radar-based human identification, demonstrating the effectiveness of micro-Doppler spectrogram features [6,7,8]. Bai et al. proposed a dual-branch network with short- and long-window Short-Time Fourier Transform (STFT) to model limb and torso motion features separately [14]. Yoon et al. developed a Dual-Scale Doppler Attention (DSDA) model to differentiate fast limb movements and slow torso motions [4]. He et al. adopted a dual-branch Swin Transformer with asymmetric cross-attention to improve closed-set human identification [15].

In addition to micro-Doppler-spectrogram-based methods, radar point-cloud processing and multimodal fusion schemes provide complementary solutions for gait recognition. Multiple-input multiple-output (MIMO) radar arrays can generate sparse point clouds containing 3D coordinates, radial velocity, and signal intensity, which characterize human geometry and spatial motion trajectories. Xue et al. exploited multi-dimensional point cloud attributes to achieve fine-grained spatiotemporal modeling [10]. Dang et al. proposed PGGait to enhance gait representation through multidimensional mmWave radar point-cloud feature fusion [5]. Wang et al. conducted point cloud-based gait recognition using an improved C3D network combined with a channel attention mechanism [16]. In terms of multi-modal fusion, Ma et al. integrated 4D radar point cloud sequences with micro-Doppler signatures to achieve feature complementarity and enhancement [9]. Han et al. proposed mmReID, a mmWave radar-based person re-identification system that feeds time-velocity micro-Doppler heatmaps into a two-stream feature-fusion network for cross-scenario pedestrian matching [17]. Ding et al. investigated robust gait recognition adaptable to multi-view and multi-clothing conditions [18]. Furthermore, studies on lightweight radar-camera fusion, distributed MIMO sensing, and video-driven mmWave point-cloud synthesis provide insights into non-radial observation and data-scarcity challenges [19,20,21].

Despite these advances, edge deployment remains a major challenge for practical radar-based gait recognition. Mainstream deep learning models often have large parameter counts and high computational overhead, limiting their deployment on resource-constrained edge devices. For instance, the classic VGG-16 baseline model contains 138 million parameters [6], while the dual-branch Swin Transformer model involves approximately 37.28 million parameters [15], making direct deployment on resource-constrained edge processors difficult. To meet the requirements of edge scenarios, lightweight radar recognition algorithms have become an active research area. Representative works include MuST-Net for real-time FMCW radar spectrogram recognition [22] and OG-PCL, which processes sparse radar point clouds using occupancy-gated parallel CNN-Bi-LSTM structures [11]. In addition, studies on mmWave-radar-based real-time human activity monitoring have demonstrated the feasibility of edge deployment [23]. Nevertheless, most lightweight schemes are designed for coarse-grained activity classification, and few studies have focused on CPU-only edge validation for fine-grained human identification. Meanwhile, most lightweight models assume stable input features and do not explicitly address feature masking caused by strong torso echoes or channel-dependent response variability under unconstrained walking conditions.

In addition to edge deployment, open-set recognition has not been sufficiently investigated in lightweight radar-based identification models. In private indoor monitoring scenarios, the system must not only identify registered users accurately but also reject interference from unregistered people and pet-induced motion. However, most existing radar-based human identification algorithms are established under closed-set assumptions, with limited research focusing on open-set recognition. Mazzieri et al. improved open-set recognition performance using point cloud adversarial autoencoders and probabilistic novelty detection [24]. Yang et al. explored the coupling mechanism between known-subject identification and unknown-subject rejection in open-set gait recognition under few-shot conditions [25]. Wang et al. investigated occlusion-robust open-set human recognition methods [26]. Yoon et al. used a causal localization network to extract human-target features and locate specific targets in uncropped micro-Doppler sequences [27]. Existing open-set studies have explored generative, metric-learning, and probabilistic novelty-detection modules [24,25,26]. Although these specialized methods can improve rejection robustness, they may introduce additional training or inference components. Lightweight algorithms enabling edge deployment while rejecting both unknown individuals and pet-induced interference remain understudied.

Related studies on nonvisual radar sensing have also emphasized temporal representation learning from subtle human signals. Xie et al. proposed mamba-enhanced attention residual U-Net with multi-task learning (MARU-MTL) for continuous blood-pressure estimation from mmWave radar pulse-wave signals. The method integrates a bidirectional Mamba module into the bottleneck of an attention-based residual U-Net to model long-range temporal dependencies [28]. Although its downstream task differs from gait identification, this work provides relevant context for extracting subtle temporal patterns from radar-derived human signals. In a recent arXiv survey, Lyu et al. reviewed edge-perception frameworks that integrate sensing, communication, computation, and edge AI, with emphasis on task-oriented and resource-aware designs for next-generation networks [29]. This system-level perspective motivates the joint consideration of recognition performance and edge-side computational cost in the present radar framework.

In summary, current radar-based gait biometric systems face three major technical challenges. First, feature representations are prone to distortion. Under strong torso scattering and unconstrained walking conditions, weak limb micromotions can be masked in micro-Doppler signatures, while a single transmit-channel response may not capture all channel-dependent scattering variations. Consequently, conventional single-channel time-frequency representations may provide incomplete gait information under the evaluated conditions. Second, achieving high recognition accuracy while maintaining low computational cost remains challenging. Most existing lightweight solutions are designed for activity classification, with few CPU-only edge-side validations dedicated to human identification tasks. Third, many open-set rejection schemes rely on sophisticated subnetworks, making it difficult to integrate stranger rejection and pet-interference suppression into low-complexity models. To address these limitations, this study develops a lightweight radar gait-identification framework and evaluates it through ablation, open-set, external-dataset, and edge-deployment experiments.

To address these challenges, this paper proposes a lightweight gait-based person identification method for MIMO mmWave radar using multi-transmit-channel differential encoding. The proposed method targets small-scale identity monitoring and is designed to improve open-set interference rejection in practical edge settings. The main contributions of this paper are summarized as follows:

1. We design an adaptive feature-enhancement and multi-transmit-channel differential-encoding scheme. Gamma correction driven by the average spectrogram intensity enhances weak limb micro-motion features. Furthermore, the Tx1 response and its absolute amplitude differences from Tx2 and Tx3 are encoded into a three-channel spatiotemporal tensor. This parameter-free encoding exploits complementary channel-dependent response differences under unconstrained walking without increasing the input size or the number of learnable parameters.
2. We construct an asymmetric-pooling convolutional recurrent neural network (Asym-CRNN) tailored to radar micro-Doppler tensors. The architecture adopts a dimension-aware lightweight design that compresses the Doppler dimension from 128 to 8 through four asymmetric 2 × 1 pooling stages while preserving all temporal steps for subsequent bidirectional long short-term memory (Bi-LSTM) modeling. The five-class model contains 1.115 million trainable parameters and has a TensorFlow Lite (TFLite) storage footprint of 4.32 MB.
3. We evaluate the method across multiple scenarios and characterize its edge-deployment performance. Experiments on a self-collected dataset evaluate closed-set identification accuracy and open-set rejection of unregistered individuals and pet-induced interference. The method is further evaluated on the public IDRad dataset after retraining to assess its adaptability to external data. In addition, network inference time, per-window preprocessing time, and the steady-state throughput of a dual-core acquisition-processing pipeline are evaluated separately on a Raspberry Pi 5 platform.

The remainder of this paper is organized as follows. Section 2 elaborates on the overall system architecture, mmWave radar signal modeling, micro-Doppler feature extraction and enhancement, multi-transmit-channel differential encoding mechanism, and the lightweight Asym-CRNN model architecture. Section 3 presents dataset construction and experimental settings, including ablation studies, analyses of observation-window length and the number of registered subjects, open-set rejection tests, external-dataset evaluation, and edge-deployment validation. Section 4 concludes the work and outlines future research directions.

## 2. Methods

### 2.1. Overall Architecture

This paper proposes a lightweight gait-based person identification method using multi-transmit-channel differential encoding for MIMO mmWave radar, establishing a cascaded processing pipeline from physical-layer feature enhancement to lightweight temporal modeling. The overall pipeline consists of FMCW-MIMO radar signal modeling, micro-Doppler feature extraction at the physical layer, adaptive background-noise suppression, Gamma correction, and multi-transmit-channel differential encoding. After physical-layer preprocessing, pseudo-color three-channel spatiotemporal tensors are constructed as input to the recognition network. On this basis, an Asym-CRNN is adopted to achieve fine-grained spatiotemporal feature learning, identity classification, and open-set rejection of unknown samples while preserving temporal resolution. The overall system architecture is illustrated in Figure 1, which presents the workflow from radar echo acquisition and physical-layer feature construction to lightweight network inference and decision-making. The framework integrates domain-specific signal-processing priors with an edge-oriented lightweight model design, providing an efficient gait-recognition solution for edge scenarios.

### 2.2. FMCW-MIMO Radar Signal Model

The proposed system adopts FMCW mmWave radar sensors for signal acquisition. Let B denote the periodic transmission bandwidth of the linear FMCW signal, $f_{c}$ denote the carrier frequency, $T_{c}$ denote the duration of a single chirp period. The initial radio-frequency waveform transmitted by the *m*-th antenna is expressed as:

(1) $$S_{T}(t)=A_{TX}exp\left(j\left(2\pi f_{c}t+\pi \frac{B}{T_{c}}t^{2}+\varphi_{0}\right)\right).$$

In the formula, $A_{TX}$ denotes the amplitude of the transmitted signal, $\varphi_{0}$ is the initial phase, and t indicates the continuous fast-time variable that satisfies $0\leq t\leq T_{c}$. When electromagnetic waves illuminate a walking human target, the non-rigid articulated motions of the limbs and torso produce time-varying radial micro-displacements. These dynamic displacements modulate the frequency of the radar echoes through the Doppler effect. The echo signals received by the radar antennas are subsequently demodulated, mixed, and low-pass filtered, yielding analog intermediate-frequency beat signals for each transmit-receive antenna pair, as illustrated in Figure 2.

Following the classic radar data cube representation, the acquired discrete baseband signals are modeled as three-dimensional tensors in this work. Let l denote the frame index, k denote the slow-time index for a given Tx-Rx channel after time-division multiplexing (TDM) demultiplexing, and s denote the fast-time sample index within each chirp. For the *l*-th frame data, the three-dimensional complex baseband matrix S(l,k,s) is modeled as:

(2) $$S(l,k,s)=A_{R}exp\left\{j\left[4\pi \left(\frac{B}{T_{c}c}\right)R(l,k)sT_{s}+\frac{4\pi }{\lambda }R(l,k)\right]\right\}+N(l,k,s),$$

where $A_{R}$ denotes the amplitude of the received echo signal, $T_{s}$ is the fast-time sampling interval of the analog-to-digital converter, λ represents the carrier wavelength, R(l,k) refers to the instantaneous radial range of the target at the *k*-th slow-time instant within the *l*-th frame, c is the speed of light, and N(l,k,s) characterizes system thermal noise and environmental clutter. This three-dimensional baseband matrix serves as the fundamental data carrier for subsequent physical-layer signal analysis and feature extraction procedures.

### 2.3. Micro-Doppler Feature Extraction at the Physical Layer

To extract micro-Doppler time-frequency features reflecting the articulated motion patterns of human gait from the raw intermediate-frequency data matrix, this work implements a preprocessing pipeline combining cascaded two-dimensional Fourier transforms with moving target indication (MTI). The resulting micro-Doppler spectrogram is illustrated in Figure 3. In this pipeline, range information is first extracted along the fast-time dimension; static clutter is then suppressed, and Doppler frequencies are estimated along the slow-time dimension. Finally, motion energy associated with different body components is aggregated through non-coherent integration along the range dimension. In the implementation, a Hamming window is applied to each frame along the fast-time dimension, followed by a range Fourier transform that maps the time-domain radar echoes to the range domain, as expressed below:

(3) $$R(l,k,r)=\sum_{s=0}^{N_{adc}-1}S(l,k,s)\cdot W_{range}(s)exp\left(-j2\pi \frac{sr}{N_{adc}}\right),$$

where R(l,k,r) denotes the complex signal in the range domain, r is the range bin index, $N_{adc}$ stands for the total number of ADC fast-time sampling points within a single chirp, and $W_{range}(s)$ represents the window function adopted for the range-dimension processing.

To suppress strong zero-Doppler clutter produced by stationary objects, mean subtraction filtering is adopted along the slow-time dimension of the intra-frame echo sequence. By removing the average static component of each frame, the direct-current (DC) offset induced by stationary objects is eliminated from every slow-time sampling point, thereby effectively highlighting dynamic human motion components. $R_{clean}(l,k,r)$ is defined as follows:

(4) $$R_{clean}(l,k,r)=R(l,k,r)-\frac{1}{N_{loops}}\sum_{k\prime =0}^{N_{loops}-1}R(l,k\prime ,r),$$

where $R_{clean}(l,k,r)$ denotes the complex matrix in the range domain after stationary clutter suppression, and $N_{loops}$ represents the total number of slow-time chirps accumulated within a single data frame.

For the clutter-filtered data, a Hamming window is reapplied along the slow-time dimension. A Doppler Fourier transform is performed to generate the two-dimensional complex range-Doppler matrix for subsequent motion feature analysis. It is expressed as follows:

(5) $$D(l,v,r)=\sum_{k=0}^{N_{loops}-1}R_{clean}(l,k,r)\cdot W_{doppler}(k)exp\left(-j2\pi \frac{kv}{N_{loops}}\right),$$

where D(l,v,r) denotes the two-dimensional complex range-Doppler matrix, v represents the index of the Doppler velocity bin, and $W_{doppler}(k)$ is the Hamming window function employed for slow-time dimension processing.

To aggregate the articulated motion energy of all human body components during walking, non-coherent integration is performed on the energy of the range-Doppler complex spectrum along the range axis. This dimensionality reduction operation compresses the three-dimensional radar data into a two-dimensional micro-Doppler time-frequency spectrogram $M_{raw}(l,v)$ which can be described as follows:

(6) $$M_{raw}(l,v)=\sum_{r}{\left|D(l,v,r)\right|}^{2}.$$

The resulting spectrogram adopts time l as the horizontal axis and Doppler frequency d as the vertical axis. It characterizes the distribution of instantaneous radial velocities of the torso, limbs, and other body components during walking, and forms the core feature representation for subsequent identity discrimination.

### 2.4. Feature Enhancement

The human torso exhibits a larger radar cross section (RCS) than the limb extremities. Accordingly, strong torso echoes dominate the micro-Doppler time-frequency spectrogram and overwhelm the weak micro-motion features of the limbs, resulting in severe feature masking and degraded gait representation quality. To mitigate this physically induced feature distortion, this paper proposes an adaptive Gamma correction algorithm driven by the average brightness of time-frequency maps. Prior to enhancement, the algorithm implements global maximum normalization on the micro-Doppler spectrogram of the *m*-th transmit channel, which preserves absolute energy discrepancy information across channels. The normalization is expressed as follows:

(7) $$I^{\left(m\right)}(l,v)=\frac{M_{raw}^{\left(m\right)}(l,v)}{G_{max}},$$

where $I^{\left(m\right)}(l,v)$ denotes the normalized spectrogram matrix whose energy amplitude is constrained within the interval [0, 1], and $G_{max}$ represents the global energy peak of micro-Doppler spectrograms generated by all transmit channels of the system within the current observation window.

The global average brightness of the time-frequency spectrogram is calculated, and the adaptive correction coefficient $\gamma^{\left(m\right)}$ is dynamically computed as follows:

(8) $$\gamma^{\left(m\right)}=\frac{\ln(0.5)}{\ln\left(\mu_{I}^{\left(m\right)}+\epsilon \right)},$$

where $\mu_{I}^{\left(m\right)}$ denotes the average energy proportion of the normalized time-frequency spectrogram, and ϵ is a small regularization constant that avoids zero values in the denominator and logarithm arguments. Before Gamma correction, each micro-Doppler spectrogram is normalized into the range [0, 1] using the maximum energy value within the current observation window. A small positive constant ϵ is introduced in the denominator and logarithmic terms to avoid numerical instability when the average spectrogram intensity approaches zero. After nonlinear transformation, the enhanced spectrogram values are clipped to the valid normalized range to prevent abnormal amplification of extremely low-energy background fluctuations. Based on the adaptive correction coefficient, a nonlinear power-law transformation is implemented to obtain the enhanced feature matrix, which is described as follows:

(9) $$I_{out}^{\left(m\right)}(l,v)={\left(I^{\left(m\right)}(l,v)\right)}^{\gamma^{\left(m\right)}}.$$

This mechanism compresses the dynamic range of strong echoes with low computational overhead. Meanwhile, it enhances micro-motion texture features in weak-echo regions, thereby highlighting fine-grained gait differences for subsequent identity recognition. Because nonlinear enhancement may also amplify background fluctuations, Gamma correction is applied after static clutter suppression and background-noise reduction. This processing order reduces the probability that stationary clutter or low-energy noise is enhanced as gait-related texture.

During unconstrained walking, the three transmit channels of the IWR6843AOP capture complementary micro-Doppler energy responses arising from differences in antenna position, Tx-Rx propagation paths, human-body scattering, and indoor multipath. The proposed differential encoding integrates these channel-dependent responses to enrich the gait representation. The physical antenna topology and corresponding virtual-array distribution are illustrated in Figure 4.

Based on this property, this work adopts the enhanced spectrogram of the main-channel Tx1 as the reference red channel. The green and blue feature maps are subsequently constructed through explicit cross-channel subtraction, and they are finally fused to form a three-channel spatiotemporal feature tensor. The feature tensors are described as follows:

(10) $$\begin{array}{c} R(l,v)=I_{out}^{\left(1\right)}(l,v),\\ G(l,v)=\left|I_{out}^{\left(1\right)}(l,v)-I_{out}^{\left(2\right)}(l,v)\right|,\\ B(l,v)=\left|I_{out}^{\left(1\right)}(l,v)-I_{out}^{\left(3\right)}(l,v)\right|, \end{array}$$

where R, G, B represent the channel carriers of the pseudo-color three-channel spatiotemporal tensor. $I_{out}(Tx1)$, $I_{out}(Tx2)$, $I_{out}(Tx3)$ represent the adaptively enhanced micro-Doppler feature matrices generated by transmit antennas Tx1, Tx2, and Tx3, respectively. These three channels are stacked along the depth dimension to form the pseudo-color three-channel spatiotemporal tensor. This transformation converts inter-channel response discrepancies and heterogeneous energy patterns across the transmit channels into distinguishable texture features that can be extracted by convolutional networks. Specifically, the red channel preserves the reference micro-Doppler intensity contour derived from the primary Tx1 channel, whereas the green and blue channels explicitly encode residual energy discrepancies between the auxiliary channels and the Tx1 reference channel. Notably, the entire encoding process requires no extra learnable parameters or matrix multiplications and only introduces linear computational overhead during processing, thereby achieving high efficiency and strong practicability for resource-limited edge deployment. The differential encoding exploits response discrepancies among the Tx-Rx propagation paths of the MIMO array. These discrepancies may reflect a mixture of weak limb-motion variations, channel-dependent scattering responses, and multipath-induced energy differences. By encoding the main Tx1 response together with the residual responses between Tx1 and the auxiliary transmit channels, the model is encouraged to attend to subtle inter-channel motion-related patterns that are less visible in a single-channel spectrogram.

Although taking the absolute value removes the polarity of inter-channel differences, this operation is deliberately adopted. The polarity depends on the selected reference channel and may be affected by channel-dependent antenna responses, propagation conditions, and indoor multipath. In contrast, the absolute difference retains the magnitude of the response discrepancy and provides a nonnegative and more consistent representation for subsequent feature extraction. To determine whether the discarded polarity contains additional discriminative information, we compared the proposed absolute-difference encoding with a signed-difference alternative in Section 3.3. The absolute-difference encoding achieved higher mean accuracy than the signed-difference alternative in all four evaluated observation windows and exhibited lower run-to-run variability across all four settings. We therefore retained the absolute-difference formulation because it provided a better overall balance between recognition accuracy and stability under the present antenna configuration and acquisition conditions.

Following the above processing operations, weak micro-motion textures are strengthened, and the inter-channel energy discrepancies are enhanced. The effectiveness of these feature improvements for gait identification is quantitatively evaluated in Section 3.3. This paper compares the original micro-Doppler spectrogram, the spectrogram after adaptive Gamma correction, and the fused features after differential encoding across multiple transmit channels, as shown in Figure 5. Panel (a) illustrates that strong torso echoes dominate the original spectrogram, rendering weak limb micro-motion features relatively indistinct. Panel (b) demonstrates that the texture in dark regions is enhanced after adaptive processing. Panel (c) shows that the inter-transmit-channel differences, encoded into a pseudo-color three-channel spatiotemporal tensor, provide additional discriminative cues related to channel differences for the network.

### 2.5. Lightweight Asym-CRNN Architecture

The three-channel pseudo-color spatiotemporal tensor generated by the physical-layer preprocessing module is fed into the lightweight convolutional recurrent neural network (CRNN) to complete spatiotemporal feature extraction and identity classification. The overall network architecture is shown in Figure 6. Specifically, the convolutional module extracts local time-frequency textures from micro-Doppler spectrograms, the asymmetric pooling module preferentially compresses redundant features, and the cascaded Bi-LSTM module captures the remaining temporal sequence information within fixed slices to model the temporal dependencies of gait movements. To balance feature-compression efficiency and temporal resolution retention, an asymmetric pooling strategy is introduced at the front end of the network. Large-stride downsampling is adopted along the Doppler frequency axis to compress redundant frequency-domain features and suppress residual background clutter, and unit-stride sampling is adopted along the time axis to preserve the sampling order and time granularity of short slice samples. Each time frame is first compressed into a compact semantic vector in the Doppler frequency domain, and subsequently fed into the temporal modeling unit in the original time order. This design alleviates the loss of gait phase variation and short-term micro-motion details arising from premature downsampling along the time dimension.

The temporal modeling component adopts a cascaded Bi-LSTM network to mine dynamic gait temporal characteristics. The forward long short-term memory (LSTM) branch captures motion correlations between the current frame and historical frames, while the backward LSTM branch extracts contextual constraint information from subsequent frames within a fixed observation slice. The concatenated hidden state sequences from both branches are utilized to characterize the transient variation patterns and dynamic dependencies of gait movements.

Because the backward branch uses subsequent frames within each completed observation slice, the Bi-LSTM is non-causal. The system produces one identity decision after each complete observation window, with total decision latency determined by data acquisition, preprocessing, and network inference.

For classification, the output at the final time step is used as the sequence representation and mapped to class probabilities through a fully connected layer. In this work, ablation experiments are used to select the number of convolutional kernels and recurrent units, balancing recognition accuracy and network complexity under the evaluated settings.

Unlike conventional CRNN architectures using symmetric pooling, the proposed Asym-CRNN avoids downsampling along the temporal axis. Its task-specific contribution is the coordinated use of Doppler-only compression and bidirectional temporal modeling, which preserves short-duration gait variations while limiting the feature dimensionality passed to the recurrent layers.

For reproducibility, the proposed Asym-CRNN consists of four convolution-batch normalization-rectified linear unit (Conv-BN-ReLU) blocks with asymmetric 2 × 1 pooling, followed by a two-layer Bi-LSTM with a hidden size of 128 and a dropout rate of 0.3. The classifier contains a dropout layer with a rate of 0.5 and a fully connected output layer. The input is a 3 × 128 × W micro-Doppler tensor, where W denotes the temporal length of the input slice. The final model is exported as a 4.32 MB TFLite model for edge deployment.

## 3. Experiments and Results Analysis

### 3.1. Experimental Setup and Dataset Construction

The data acquisition platform was constructed using a TI IWR6843AOP mmWave radar with 3T4R antennas integrated in an antenna-on-package configuration. The radar waveform, time-division multiplexing multiple-input multiple-output (TDM-MIMO) sequence, antenna configuration, mounting geometry, and principal acquisition parameters are summarized in Table 1. These fundamental parameters collectively determine the range resolution, velocity measurement range, and temporal sampling density of the system.

The three transmit antennas were activated sequentially in the order Tx1-Tx2-Tx3 using TDM-MIMO. Each frame contained 128 TDM loops, with three transmitted chirps per loop and 384 transmitted chirps in total. The frame period was 40 ms. The radar was mounted at a height of 2.15 m and tilted downward by approximately 15°, and the radar-to-subject distance ranged from approximately 0.8 to 6 m. According to the manufacturer, the integrated antenna layout uses a nominal wavelength of λ = 5 mm and relative element spacings of λ and λ/2. The device datasheet specifies a single-transmitter effective isotropic radiated power (EIRP) of 16 dBm and a transmitter power-backoff range of 26 dB. The configured transmitter power backoff was 0 dB; therefore, the nominal single-transmitter EIRP was approximately 16 dBm. Typical transmitter and receiver H-plane and E-plane radiation patterns are provided in the datasheet of the TI IWR6843AOP. Tx/Rx channel calibration relied on the built-in RF initialization and calibration procedures of the IWR6843AOP, without an additional reference-target calibration step. The manufacturer reports the single-transmitter EIRP and normalized antenna radiation patterns rather than a separate absolute antenna-only gain. Polarization was not specified in the available datasheet, and neither antenna-only gain nor polarization was independently measured in this study.

Targeting small-scale closed-set monitoring scenarios, such as private offices and smart-home environments, we construct a radar gait baseline dataset for real-world deployment. The experiment recruited a total of 5 subjects, including 4 males and 1 female. For each of the five registered subjects, ten independent 20 s radar echo sequences were collected, resulting in a total of 50 original acquisition sequences. These recordings were acquired on different dates and at different times, and subjects wore different daily clothing across recording sessions to introduce realistic intra-subject variations. The participants have heights ranging from 160 cm to 185 cm and weights from 50 kg to 88 kg, covering the basic physical characteristics of users in typical edge application scenarios such as small families and independent office spaces. The dataset captured variations in recording time, clothing, footwear, and natural walking conditions. During each acquisition, participants walked freely within the radar test area. The 50 original acquisition sequences were divided into 35 training sequences, 10 validation sequences, and 5 test sequences, corresponding to 7, 2, and 1 sequences per subject, respectively. The dataset was split at the original acquisition-sequence level before receiver-wise expansion and temporal slicing. Each original 20 s recording was treated as an indivisible group, and all Rx-channel observations, temporal slices, and augmented variants derived from the same recording were assigned exclusively to the same subset. Receiver-wise expansion and temporal slicing were subsequently performed separately within each subset, yielding 40 slices per receive channel per recording, i.e., 1600 derived channel-wise samples per registered subject and 8000 in total across the 50 recordings. The experimental scenarios are illustrated in Figure 7, which includes two typical environments: free walking of subjects and pet-induced dynamic interference. Such a setup supports both closed-set identity recognition and open-set outlier rejection experiments. All subjects provided signed informed consent before participating in the data collection campaign.

### 3.2. Evaluation Metrics and Baseline Comparisons

Experiments were conducted to evaluate recognition accuracy, model compactness, computational complexity, and edge-side processing performance. To quantitatively benchmark the performance boundaries of the proposed architecture, two classic deep learning models are adopted as comparative baselines. For a fair structural comparison, all closed-set recognition experiments utilize physically enhanced three-channel spatiotemporal tensors as inputs for the standard CNN baseline, standard ResNet-18, and the proposed CRNN model, except for the single-channel data adaptation experiment of IDRad. All compared models were trained and evaluated using the same acquisition-sequence-level train/validation/test split. The same sequence-level grouping was preserved after receiver-wise sample expansion, ensuring that Rx-channel samples from the same original recording were never distributed across different subsets. The validation set was used only for model selection, whereas the test set remained isolated for final performance reporting. The maximum softmax probability (MSP) threshold was set to 0.85. To account for variability in model training, each experimental setting was independently trained five times using different random seeds while maintaining the same fixed data split. The resulting accuracies are reported as the mean ± standard deviation across the five runs. The proposed CRNN was trained using the Adam optimizer with an initial learning rate of 5 × 10^−4^, a weight decay of 1 × 10^−3^, and a batch size of 32 for 60 epochs. Cross-entropy loss was used for supervised identity classification. An exponential learning-rate scheduler with a decay factor of 0.96 was applied after each epoch. Data augmentation, including additive Gaussian noise, Doppler-axis shifting, and temporal scaling, was applied only to the training set, whereas the validation and test sets remained unaugmented.

### 3.3. Ablation Study

Ablation experiments on the real-world closed-set dataset were performed to quantify the accuracy improvements brought by each designed module. All ablation results in Table 2 were obtained under the acquisition-sequence-level split described above, so slices from the same 20 s recording did not cross the training-test boundary. All four conditions in Table 2 used the same Asym-CRNN classification backbone, data split, optimizer, training schedule, and repeated-run protocol. Only adaptive Gamma correction and transmit-channel differential encoding were enabled or disabled. When differential encoding was disabled, the Tx1 spectrogram was used as the network input. Therefore, the unenhanced baseline in Table 2 refers to the Asym-CRNN operating on the original Tx1 input. The results regarding the efficacy of the physical-layer feature enhancement pipeline are summarized in Table 2, where ‘×’ and ‘√’ denote the deactivation and activation of the corresponding module, respectively. Four groups of comparative experiments were conducted to validate the individual and combined effects of the proposed enhancement strategies. The results showed that each module improved the mean recognition accuracy under the fixed Asym-CRNN backbone, and that their combination achieved the highest mean accuracy. Relative to the unenhanced Tx1 baseline, which achieved 85.58% and 91.03% for the 1 s and 6 s windows, respectively, the combined configuration achieved 89.62% and 99.52%. These findings support the complementary contributions of adaptive Gamma correction and transmit-channel differential encoding under the evaluated dataset and fixed data split. From a physical-mechanism perspective, the two modules optimize radar micro-motion features from complementary dimensions and provide complementary improvements in gait-feature representation. Adaptive Gamma correction mainly recovers weak limb micro-motion patterns masked by dominant torso echoes. It enhances the saliency of low-intensity micro-motion textures, thereby improving the inter-class distinguishability of single-channel gait features. The independent contribution of adaptive Gamma correction is also reflected in the ablation results. Without transmit-channel differential encoding, Gamma correction improves the 1 s accuracy from 85.58% to 86.67% and the 6 s accuracy from 91.03% to 96.47%. These improvements indicate that adaptive nonlinear enhancement strengthens weak limb-related micro-Doppler textures while maintaining recognition stability. In contrast, multi-transmit-channel differential encoding exploits inter-channel residual energy discrepancies across multiple Tx-Rx propagation paths. This encoding exposes channel-dependent motion patterns and complementary discriminative cues that are less visible in the single-channel micro-Doppler representation. The ablation results provide quantitative support for the contribution of transmit-channel differential encoding. Without Gamma correction, introducing channel differential encoding improved the 1 s and 6 s accuracies from 85.58% to 86.83% and from 91.03% to 95.45%, respectively. This indicated that inter-channel residual information provided complementary discriminative cues beyond the single-channel micro-Doppler representation. These two modules provided single-channel feature enhancement and inter-channel response complementarity, yielding performance gains for radar-based gait recognition. The accuracy values in Table 2, Table 3, Table 4 and Table 5 represent averaged results over five repeated training runs with different random seeds, rather than a single stochastic training outcome.

To distinguish the contribution of the differential operation from the benefit of using multiple transmit channels, we further compared four input-encoding strategies: Tx1-only input, direct stacking of the Tx1-Tx2-Tx3 observations, signed inter-channel differences, and the proposed absolute-difference encoding. The same Asym-CRNN backbone and training protocol were used for all four conditions, such that only the transmit-channel input representation was changed. The 80.40% standard CNN result reported in Section 3.4 evaluates a different experimental factor: all network architectures in that comparison receive the same fully enhanced three-channel tensor, while only the classification backbone is changed. These values correspond to different comparisons: Table 2 varies the input enhancement under a fixed Asym-CRNN, whereas Section 3.4 varies the network backbone under the same enhanced input.

Adaptive Gamma correction was applied identically to all four input representations. The same data split, Asym-CRNN architecture, training protocol, and random-seed settings were used, so only the transmit-channel representation was varied.

As shown in Table 3, direct Tx stacking consistently outperformed the Tx1-only input, confirming that the use of multiple transmit channels itself provides additional discriminative information. The proposed absolute-difference encoding achieved higher mean accuracy than direct Tx stacking in all four evaluated observation windows. It also outperformed the signed-difference alternative in mean accuracy at all four window lengths and exhibited lower standard deviations than the signed-difference representation in every setting. Encoding the magnitude of the inter-channel discrepancy provided higher accuracy and greater stability than retaining its polarity. Therefore, the absolute-difference encoding was retained as the proposed formulation.

Table 4 presents the ablation results for the spatiotemporal network architecture. All network variants were evaluated using the same fixed split and the same physically enhanced three-channel input. The 1 s observation window was selected because short-duration inputs are particularly sensitive to the loss of temporal detail. The comparison isolates two architectural choices: asymmetric Doppler-only pooling and Bi-LSTM-based temporal modeling. Experimental results reveal that the conventional Sym-CNN with symmetric pooling introduces premature temporal downsampling. This operation eliminates abundant short-term gait-phase information and subtle micro-motion features, restricting its recognition accuracy to only 83.62%. By adopting asymmetric pooling, the Asym-CNN network preserves fine-grained temporal features, thereby improving the accuracy to 86.75%. On this basis, cascading a Bi-LSTM to extract bidirectional temporal contexts further elevates the accuracy of the Asym-CRNN to 89.62%. This performance gain indicates that the asymmetric pooling and Bi-LSTM modules complement each other effectively, jointly enhancing the capability to resolve gait features from short-duration slices.

### 3.4. Effects of Observation Window Length and Number of Registered Subjects

In complex indoor scenarios with multipath reflection interference, the duration of the radar observation window directly determines the integrity of the captured gait-cycle information. Figure 8 illustrates the micro-Doppler spatiotemporal feature maps corresponding to observation windows ranging from 1 s to 6 s. It can be observed that extending the observation window incorporates more complete gait cycles and enriches the periodic texture characteristics generated by limb motions. This intuitive feature variation provides a reasonable physical explanation for the positive correlation between recognition accuracy and observation window length in subsequent quantitative experiments.

The recognition accuracy of different network architectures under observation windows of varying durations is shown in Figure 9. For a controlled architectural comparison, the standard CNN, ResNet-18, and the proposed CRNN adopt identical physically enhanced three-channel spatiotemporal tensors as network inputs. This unified input configuration reduces confounding from differences in data preprocessing, enabling a focused evaluation of the inherent feature-extraction capabilities of diverse network topologies. Under short observation windows of 1 s and 2 s, the proposed CRNN achieved average recognition accuracies of 89.62% and 95.87%, respectively, which were higher than the corresponding CNN baseline results with comparable parameter scales. The CNN only obtained accuracies of 80.40% and 91.52% under the same experimental settings. Furthermore, the proposed CRNN also outperformed the standard ResNet-18, which has more parameters and a more intricate structural design. Specifically, ResNet-18 achieved accuracies of 87.51% and 94.17% for 1 s and 2 s observation samples, respectively. When the observation window length was extended to 6 s, the CRNN achieved a mean accuracy of 99.52%. Such a performance was competitive with that of the deeper ResNet-18 network. Meanwhile, the proposed method reduced network redundancy while maintaining competitive recognition accuracy under the evaluated settings.

The effects of the number of registered subjects on recognition accuracy are presented in Table 5. For each registered-subject setting, the same split protocol was applied to avoid overlap between training and testing slices derived from the same recording. To evaluate the recognition robustness of the proposed system for small-scale closed-set monitoring applications, the baseline number of registered subjects is progressively increased in the experiments. The recognition accuracy trends under both short and long observation windows are compared as the number of subjects increases from 2 to 5. The experimental results show that, with the growth of registered subjects, the system accuracy under the 1 s short-term data drops steadily from 98.12% to 89.62%. This performance degradation can be attributed to the limited discrimination of instantaneous micro-motion features contained in short-duration samples. As the number of identity classes increased, greater inter-class similarity may have contributed to the reduction in short-window recognition accuracy. In contrast, the system maintains prominent robustness under the 6 s long-term data. Even when the number of registered subjects expands to 5, the recognition accuracy remains as high as 99.52%. These results suggest that longer observation windows provide more complete temporal gait information under the evaluated small-cohort conditions. The enriched temporal feature representation significantly improves the recognition stability in multi-identity closed-set scenarios, enabling reliable high-precision gait recognition for practical indoor monitoring environments. These results characterize the effect of cohort size within the evaluated range of two to five registered subjects.

### 3.5. Open-Set Rejection Capability Evaluation

In smart homes and unobtrusive indoor monitoring scenarios, dynamic disturbances including indoor pet activities and unknown stranger intrusions trigger false identity recognition outputs, which severely degrade system monitoring reliability. To tackle this issue, two groups of open-set rejection experiments are conducted to evaluate the capability of the model to identify and exclude unknown interference samples. Figure 10 presents typical micro-Doppler spectrograms of a freely walking pet cat. Different from the continuous and periodic swinging texture of human gait, pet radar echoes exhibit distinct high-frequency and short-period pulsating characteristics. Such feature discrepancies are further magnified after physical-layer feature enhancement, yielding highly distinguishable time-frequency patterns. The pet-interference dataset comprised two cats recorded over multiple acquisition sessions; after quality screening, a total of 270 s of valid radar data was retained (170 s and 100 s for the two animals). The pet recordings and their derived slices formed an independent test set, while model training and threshold setting used the human dataset. All 89 pet slices generated under the 3 s setting and all 44 pet slices generated under the 6 s setting were correctly rejected. This rejection rate was obtained for the two cats and the acquisition sessions evaluated in this study.

To further validate the discriminative capability against highly similar unknown intruders, additional open-set rejection experiments were carried out for unregistered individuals. The test dataset was constructed by integrating 160 gait slices collected from three unregistered individuals into the original 6 s closed-set test dataset. This mixed test set was used to evaluate both the rejection of unknown individuals and the retention of registered residents under the same decision threshold. The three unregistered individuals were not involved in model training. The MSP threshold was set to 0.85 for the open-set evaluation. Using MSP as the confidence score for registered-subject membership, the threshold-independent area under the receiver operating characteristic curve (AUROC) calculated from 800 registered-subject slices and 160 slices from three unregistered individuals was 0.9869. At an MSP threshold of 0.85, 147 of 160 unregistered samples were rejected, and 13 were incorrectly accepted, yielding a false acceptance rate (FAR) of 8.12%, while 1 of 800 registered samples was rejected, yielding a false rejection rate (FRR) of 0.12%. Accordingly, the unregistered-subject rejection rate and registered-subject acceptance rate were 91.88% and 99.88%, respectively. The overall six-class open-set accuracy was 98.33%. Here, FAR denotes the proportion of unregistered samples accepted as registered, whereas FRR denotes the proportion of registered samples rejected as unknown. These slice-level FAR and FRR values summarize the present cohort, with multiple slices grouped by their source acquisition sequences. The confusion matrix is shown in Figure 11.

MSP was adopted as a lightweight, parameter-free rejection rule that directly reuses the classifier output. It requires no auxiliary generative model or metric-learning branch and adds negligible inference cost.

### 3.6. Adaptability Validation on External Datasets and Comparison with Existing Methods

Notably, model training and testing were performed on the public IDRad dataset. All experiments adopted dataset-specific retraining on IDRad to deliver an external-dataset evaluation of the proposed architecture.

Using 3 s inputs under a unified data-partitioning protocol, the baseline CNN achieved a recognition accuracy of 76.80%. Adaptive Gamma correction increased the accuracy to 82.70%, achieving a 5.90-percentage-point improvement over the baseline. Further incorporating temporal modeling through the proposed Asym-CRNN increased the accuracy to 86.98%, which outperformed the Gamma-enhanced CNN by 4.28 percentage points and delivered a cumulative accuracy gain of 10.18 percentage points relative to the original baseline. These results show that adaptive Gamma correction and asymmetric-pooling temporal modeling extract identity-discriminative features from the single-channel IDRad micro-Doppler data.

To contextualize the external-validation results, Table 6 summarizes representative radar-based gait-identification studies with broadly comparable cohort sizes. Vandersmissen et al., Yoon et al., and Feng et al. evaluated their methods on the IDRad dataset, providing useful reference points for evaluating our method on this public dataset. Dang et al. evaluated a method based on multidimensional millimeter-wave radar point clouds for gait identification in a small cohort. Because these studies differ in input representation and evaluation protocol, their results are included to contextualize different technical approaches rather than to support direct performance ranking.

As shown in Table 6, Vandersmissen et al. reported an identification accuracy of 78.46% on IDRad using a 3 s observation window, whereas our method achieved 86.98% under the same nominal window length. Yoon et al. reported 91.35% using a 10 s window, and Feng et al. achieved 84.67% using 6.5 s micro-Doppler inputs with an optoelectronic reservoir-computing model. These comparisons suggest that the proposed method can extract identity-discriminative information from relatively short radar observations. However, because the compared studies differ in data partitioning, preprocessing, model optimization, and evaluation protocols, these results should be interpreted as contextual evidence rather than a rigorous head-to-head ranking. A controlled comparison would require a common data split, consistent metrics, leakage-free evaluation, and fair model optimization. Dang et al. reported 96.75% using sparse 5D radar point clouds from nine subjects in a single-person closed-set setting, which provides complementary context because of its distinct input modality and evaluation design.

### 3.7. Edge Deployment Latency and Lightweight Network Inference Analysis

The five-class Asym-CRNN contains 1,114,821 trainable parameters. Computational complexity was profiled with a batch size of one. For the 1 s input tensor of 3 × 128 × 25, the model requires 65.38 MMACs, corresponding to approximately 130.77 MFLOPs when multiplication and addition are counted separately. For the 6 s input tensor of 3 × 128 × 150, the complexity increases to 392.30 MMACs, or approximately 784.59 MFLOPs. The parameter count remains unchanged across observation windows, while the computational cost increases approximately linearly with temporal length.

To evaluate edge-side computational performance, the acquisition and processing pipeline was deployed on a Raspberry Pi 5 using CPU-only execution, with hardware accelerators disabled. One CPU core was dedicated to radar-data reception and buffering, whereas a second CPU core processed the previously completed observation window. A double-buffering mechanism allowed reception of window *k* + 1 to overlap with preprocessing and inference for window *k*. Table 7 therefore reports the processing demand for each completed window, decomposed into preprocessing time, network inference time, and their sum.

For the 1 s input, signal preprocessing consumes 709 ms and network inference 6.7 ms, resulting in a total per-window processing time of 715.7 ms. For the 6 s input, preprocessing and network inference took 4351 ms and 15.2 ms, respectively, giving a total per-window processing time of 4366.2 ms. In the implemented pipeline, acquisition of window *k* + 1 proceeded concurrently with processing of window *k*. The cold-start time from the beginning of the first observation to the first identity output was therefore approximately 1.716 s for the 1 s setting and 10.366 s for the 6 s setting. After pipeline warm-up, the steady-state output interval is determined by maxmax(*T*_obs_, *T*_proc_). Because 715.7 ms is shorter than 1 s and 4366.2 ms is shorter than 6 s, the pipeline produced one result per non-overlapping observation window without processing backlog under the evaluated single-target condition. The corresponding processing margins were 284.3 ms and 1633.8 ms, respectively. The measured processing margins provide headroom for future evaluation of overlapping windows, multiple targets, and additional background workloads.

The per-window CPU processing time was dominated by signal-preprocessing operations such as FFT transformation and cross-channel differential encoding, whereas neural-network inference accounted for only a small fraction of the measured processing time. In this work, the term lightweight refers specifically to the parameter count of the classification backbone, the storage footprint of the exported model, and CPU-only neural-network inference time on embedded hardware.

## 4. Discussion

To address privacy concerns and the limited computational resources of edge devices for smart homes, private offices, and non-intrusive indoor monitoring scenarios, this work proposes a lightweight gait recognition scheme based on multi-transmit-channel differential encoding of MIMO millimeter-wave radar micro-Doppler signatures for edge deployment. By co-designing the physical-layer feature enhancement pipeline and a lightweight temporal neural network, the proposed method stabilizes discriminative time-frequency features and tailors the classification backbone for efficient edge execution.

At the physical signal preprocessing stage, an adaptive Gamma correction module is developed to dynamically adjust transformation parameters according to the average intensity of micro-Doppler spectrograms. This parameter-free enhancement strategy mitigates the masking effect of dominant torso echoes on faint limb micro-motion textures without introducing additional learnable parameters; its runtime cost is included in the preprocessing time reported in Table 7. Furthermore, the multi-transmit-channel differential encoding scheme exploits channel-dependent response discrepancies among the 3T4R propagation paths. By combining the Tx1 reference response with the absolute residuals between Tx1 and Tx2/Tx3, the proposed encoding provides complementary gait features without requiring additional learnable parameters. On the network architecture side, the asymmetric pooling CRNN compresses redundant frequency-domain features along the Doppler axis while fully preserving fine temporal granularity of short segmented samples. Cascaded Bi-LSTM layers are incorporated to model bidirectional long-range temporal dependencies embedded within gait sequences. The exported TFLite classification model occupies 4.32 MB of storage, indicating a small network memory footprint suitable for resource-constrained edge devices. Preprocessing accounts for most of the end-to-end latency and is the primary target for further optimization.

Extensive ablation, comparative, and open-set experiments demonstrate the effectiveness of the proposed framework under the tested small-cohort indoor settings. Within a small-scale indoor closed-set scenario covering five registered participants and using acquisition-sequence-level train/test separation, the system achieves average recognition accuracies of 89.62% and 99.52% for the 1 s and 6 s observation windows, respectively. For open-set evaluation, the model rejected all evaluated pet slices and 147 of 160 unregistered-person slices (91.88%). For registered-versus-unregistered discrimination, the AUROC was 0.9869. At an MSP threshold of 0.85, the FAR and FRR were 8.12% and 0.12%, respectively. The overall six-class open-set accuracy was 98.33%. External-dataset validation after retraining on the public single-channel IDRad dataset yielded an accuracy of 86.98% without multi-channel differential enhancement. On the Raspberry Pi 5, network-only inference remained below 20 ms, while the total per-window processing times were 715.7 ms and 4366.2 ms for the 1 s and 6 s inputs, respectively. The dual-core implementation overlapped processing of one completed window with acquisition of the next window. Because the processing time was shorter than the corresponding observation duration, the steady-state output interval matched the non-overlapping window duration.

The experimental results show that the proposed approach provides a practical balance between recognition accuracy and model compactness under the evaluated five-subject setting, while the per-window CPU processing time remains dominated by preprocessing. These results suggest the potential applicability of the proposed method to privacy-sensitive, contactless identity monitoring in small-scale indoor environments. The present evaluation focuses on five registered participants and the tested pet and unregistered-person interference conditions. The dual-core pipeline maintained real-time throughput for non-overlapping single-target windows, with processing margins of 284.3 ms and 1633.8 ms. The five repeated runs quantify training variability under a fixed acquisition-sequence split. Future work will evaluate variability across additional session partitions and larger participant cohorts.

## Figures and Tables

**Figure 1:**
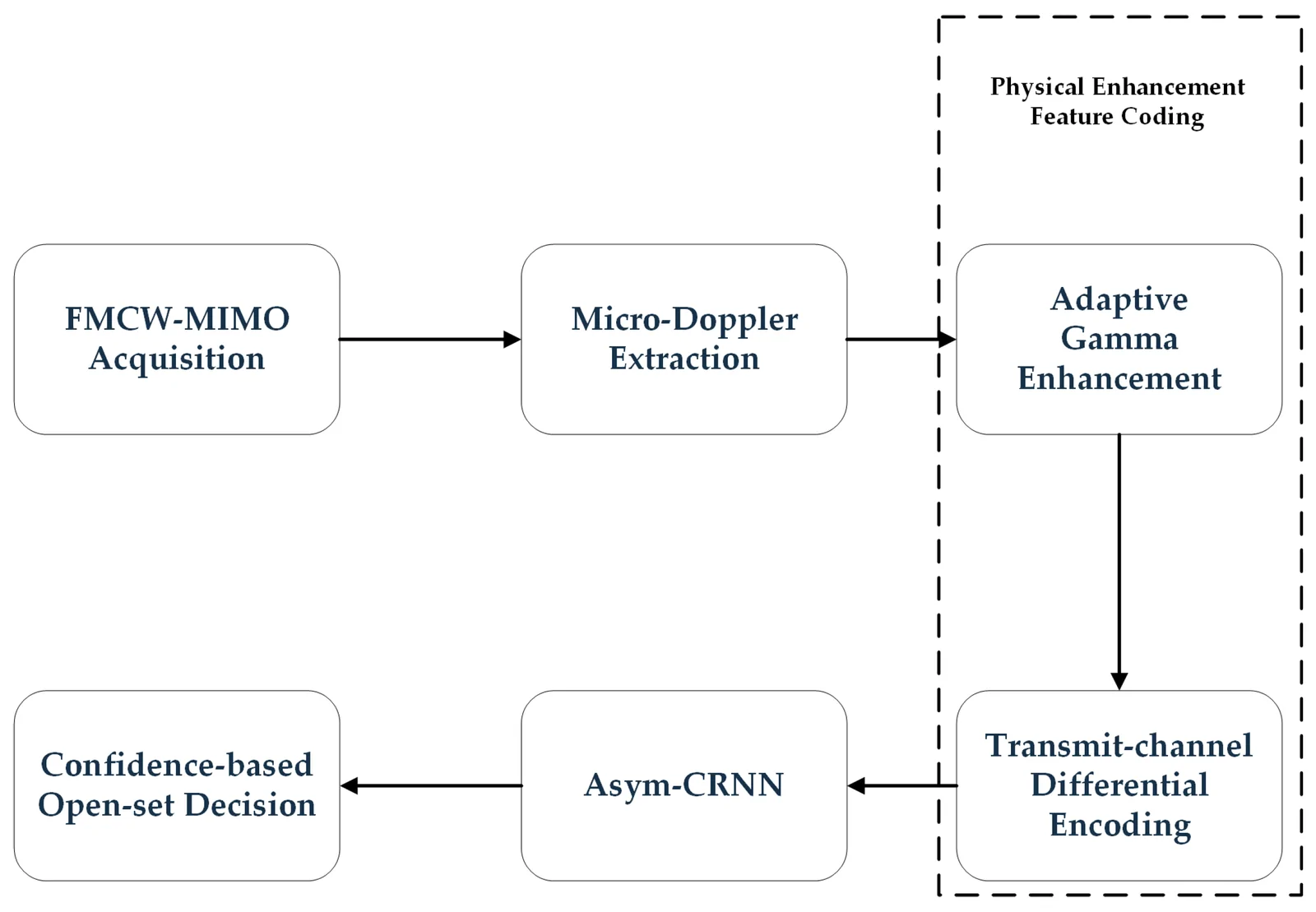
Overall architecture of the lightweight gait recognition system based on MIMO mmWave radar.

**Figure 2:**
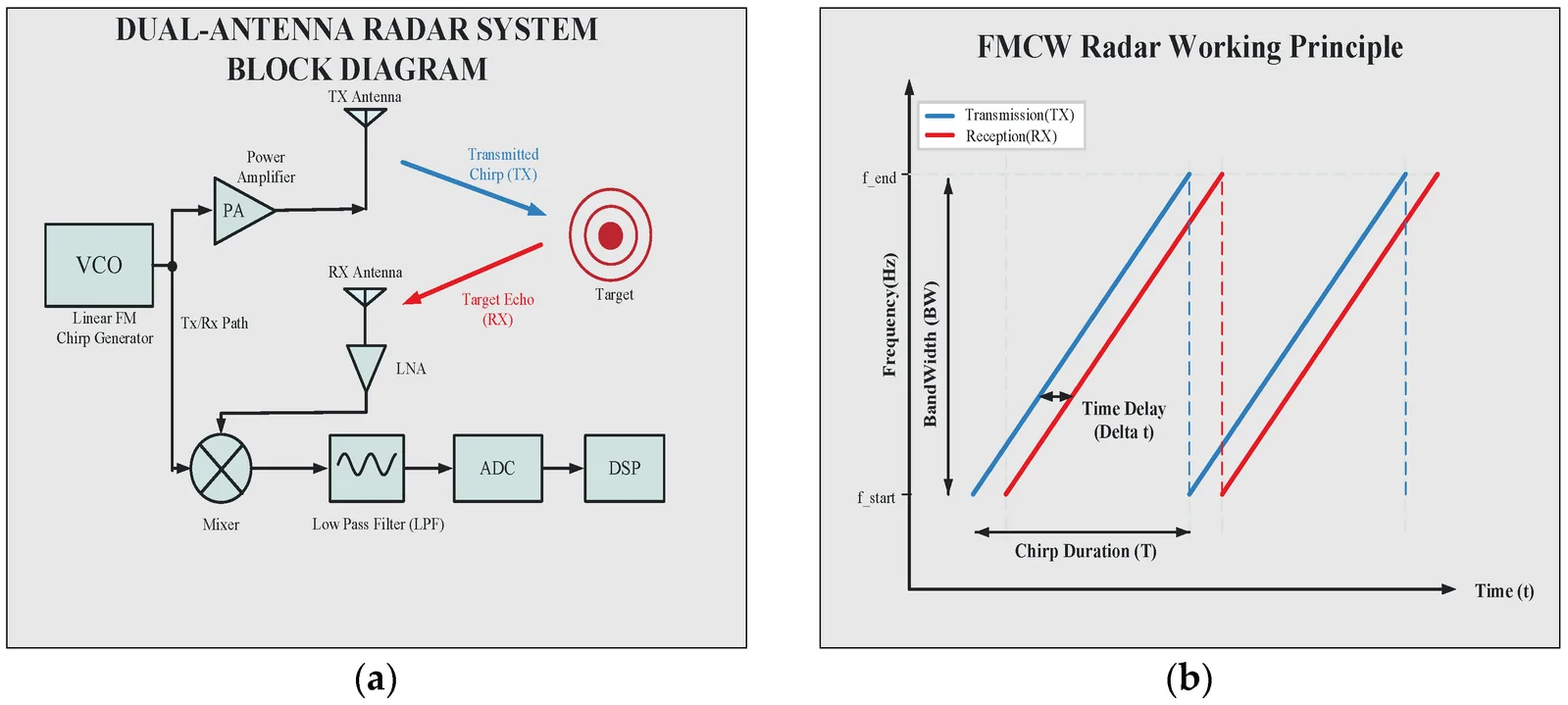
FMCW radar signal transmission, reception, and intermediate-frequency beat-signal generation: (**a**) Typical RF front-end chain of a single-transmit, single-receive radar; (**b**) Linear frequency-time relationship of FMCW chirp signals.

**Figure 3:**
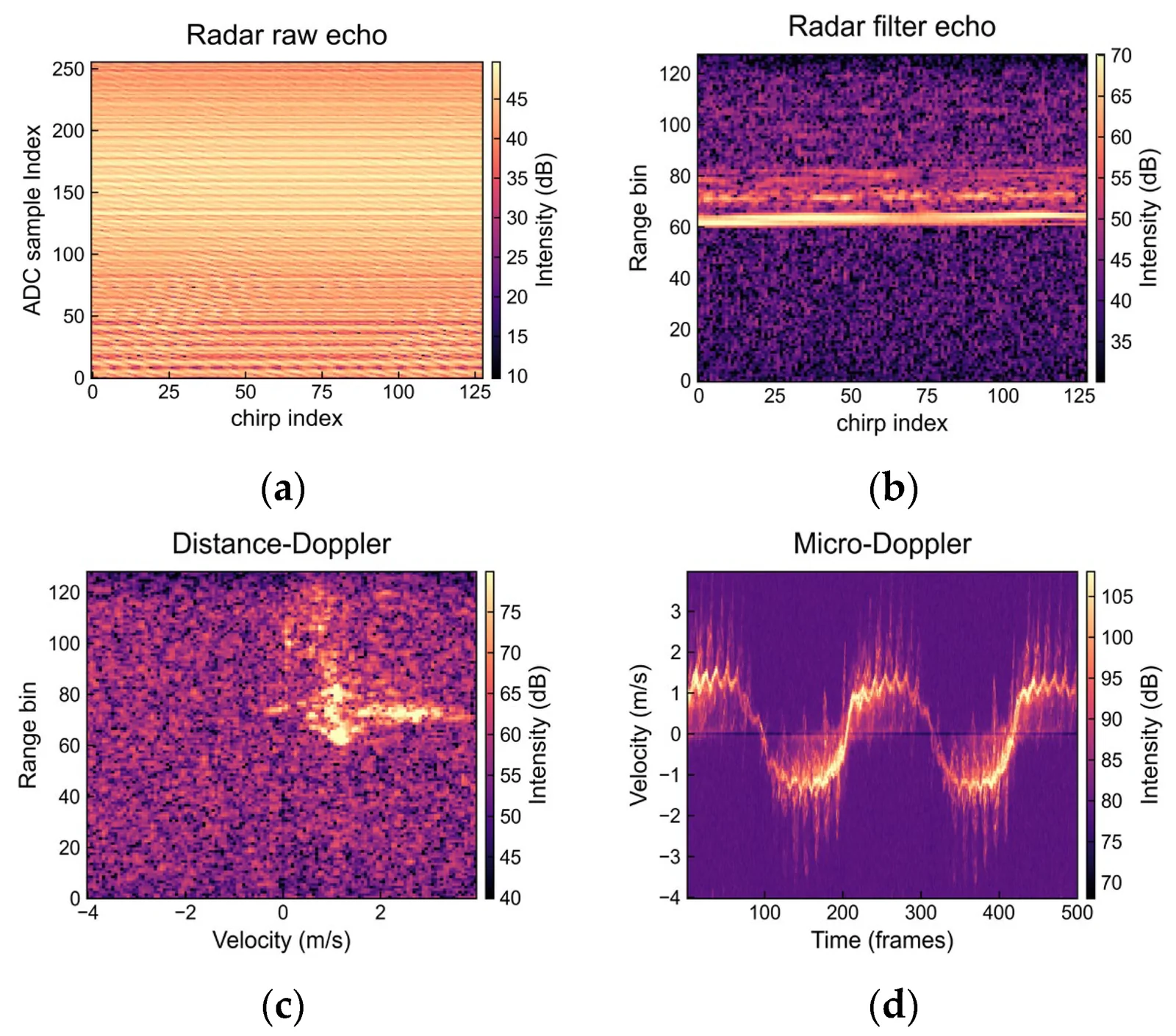
Radar-echo preprocessing and micro-Doppler spectrogram generation: (**a**) Raw range-FFT echoes derived from the ADC data; (**b**) Range-FFT echoes after MTI clutter suppression; (**c**) Two-dimensional range-Doppler spectrum obtained by 2D FFT; (**d**) Micro-Doppler time-frequency spectrogram obtained through non-coherent integration along the range dimension.

**Figure 4:**
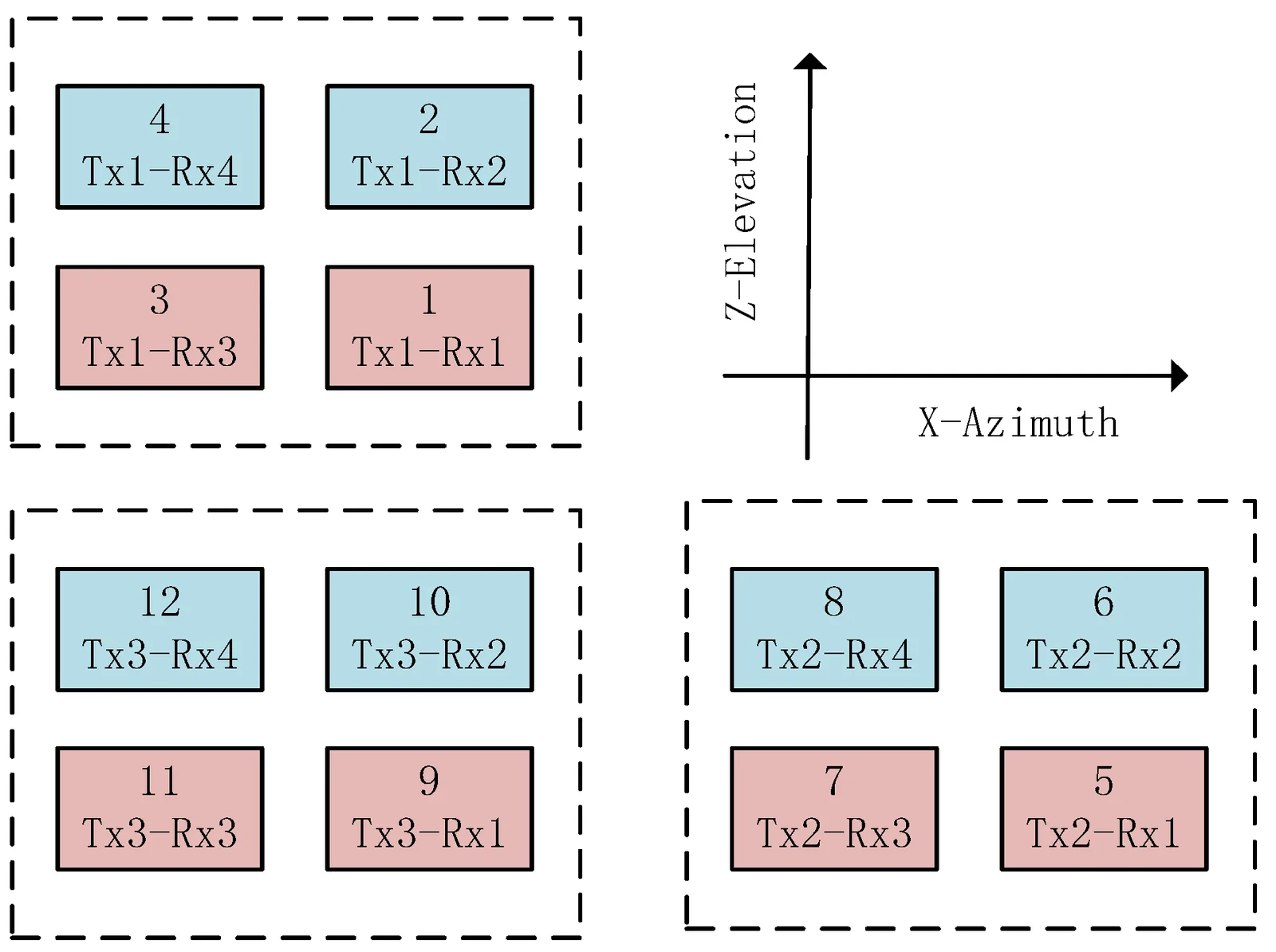
Physical topology and equivalent virtual element layout of a three-transmit four-receive (3T4R) MIMO mmWave radar antenna array.

**Figure 5:**
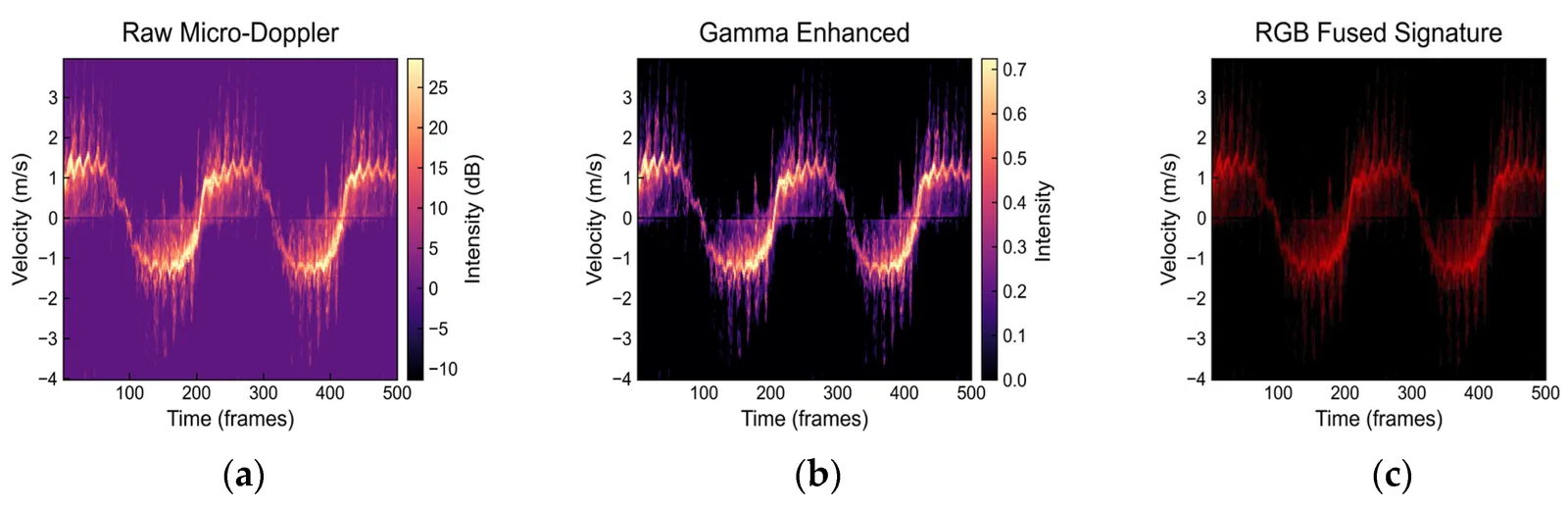
Comparison of micro-Doppler feature enhancement and multi-transmit-channel differential encoding effects. (**a**) Raw micro-Doppler spectrogram obtained via non-coherent energy integration along the range axis; (**b**) Micro-Doppler spectrogram after adaptive background noise suppression and Gamma correction; (**c**) Pseudo-color three-channel spatiotemporal tensor after multi-transmit-channel differential encoding.

**Figure 6:**
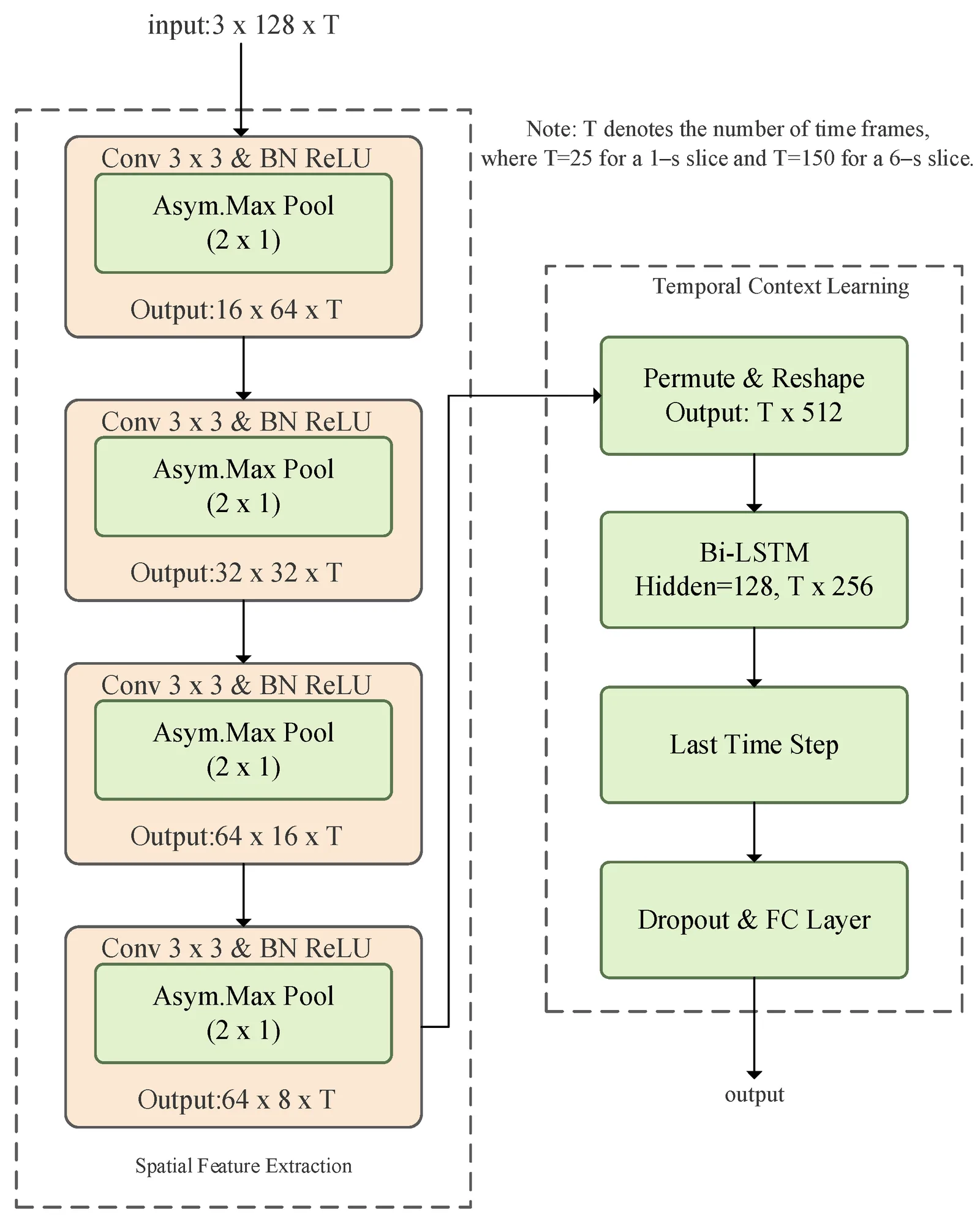
Lightweight CRNN spatiotemporal feature extraction architecture integrating asymmetric pooling and Bi-LSTM.

**Figure 7:**
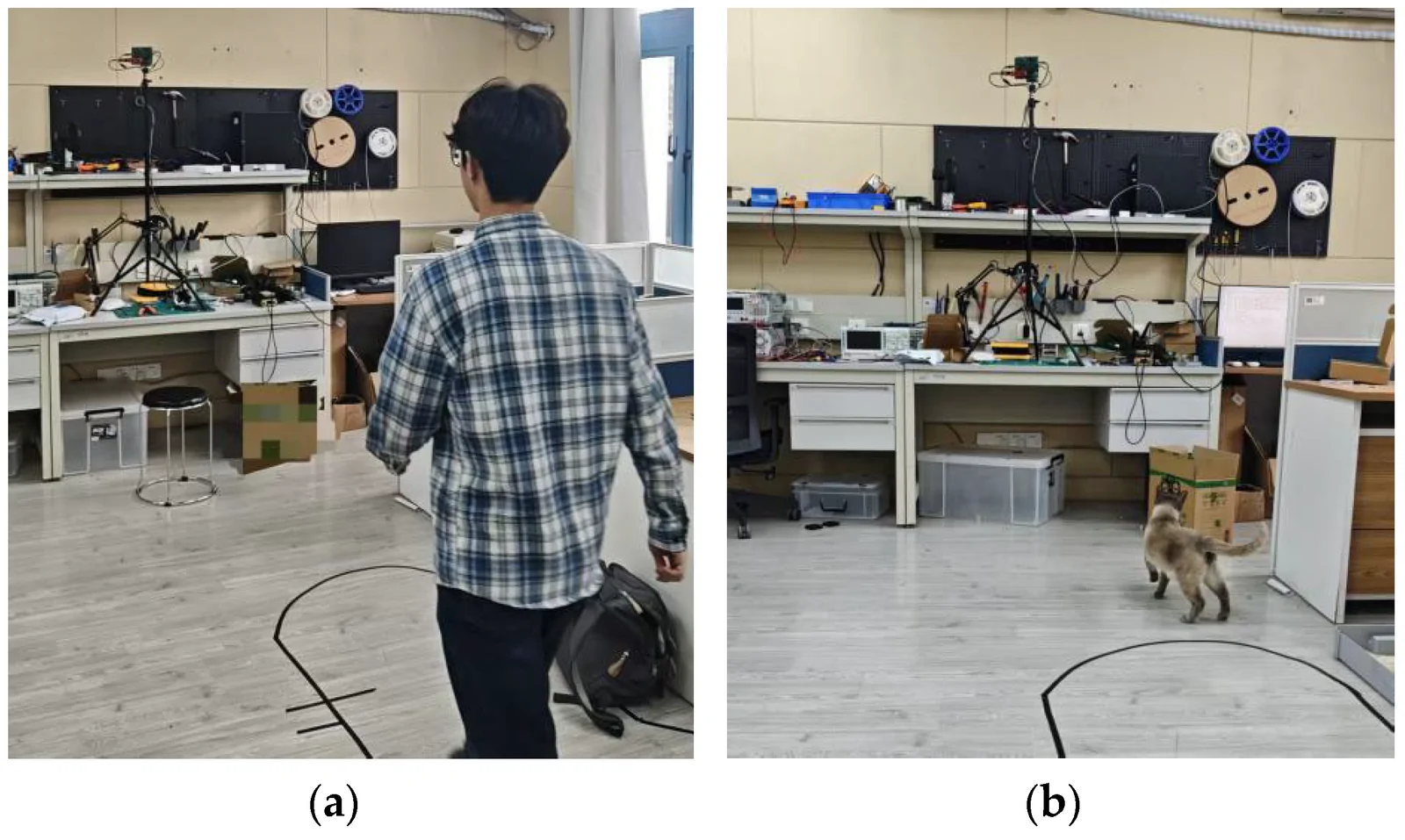
Data acquisition scenario in an indoor environment with strong multipath effects. (**a**) The subject walks freely without constraints within the radar line of sight; (**b**) Dynamic interference scenario caused by a pet cat during data acquisition.

**Figure 8:**
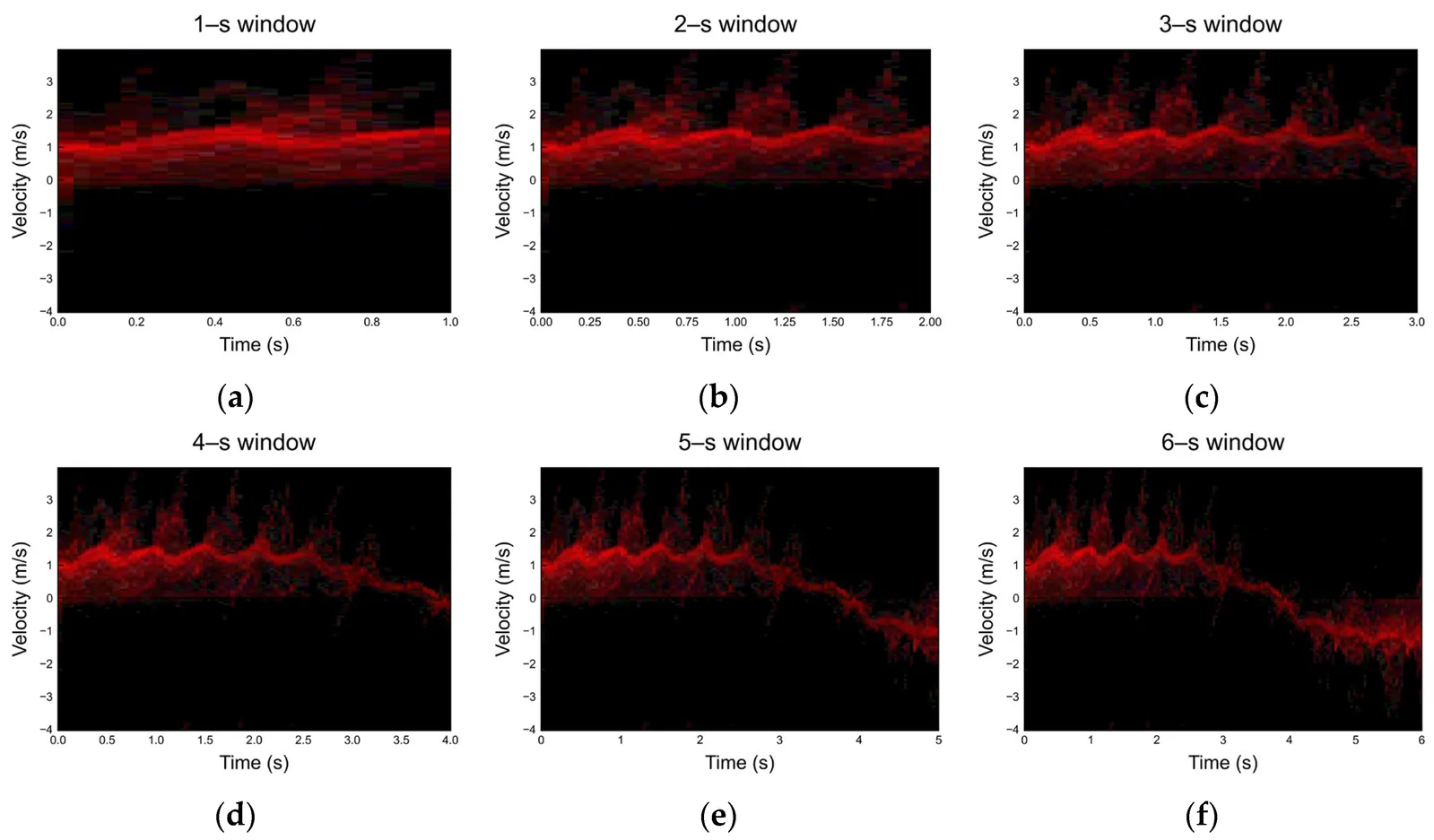
Micro-Doppler spatiotemporal feature maps under different observation windows. (**a**) 1–s window; (**b**) 2–s window; (**c**) 3–s window; (**d**) 4–s window; (**e**) 5–s window; (**f**) 6–s window.

**Figure 9:**
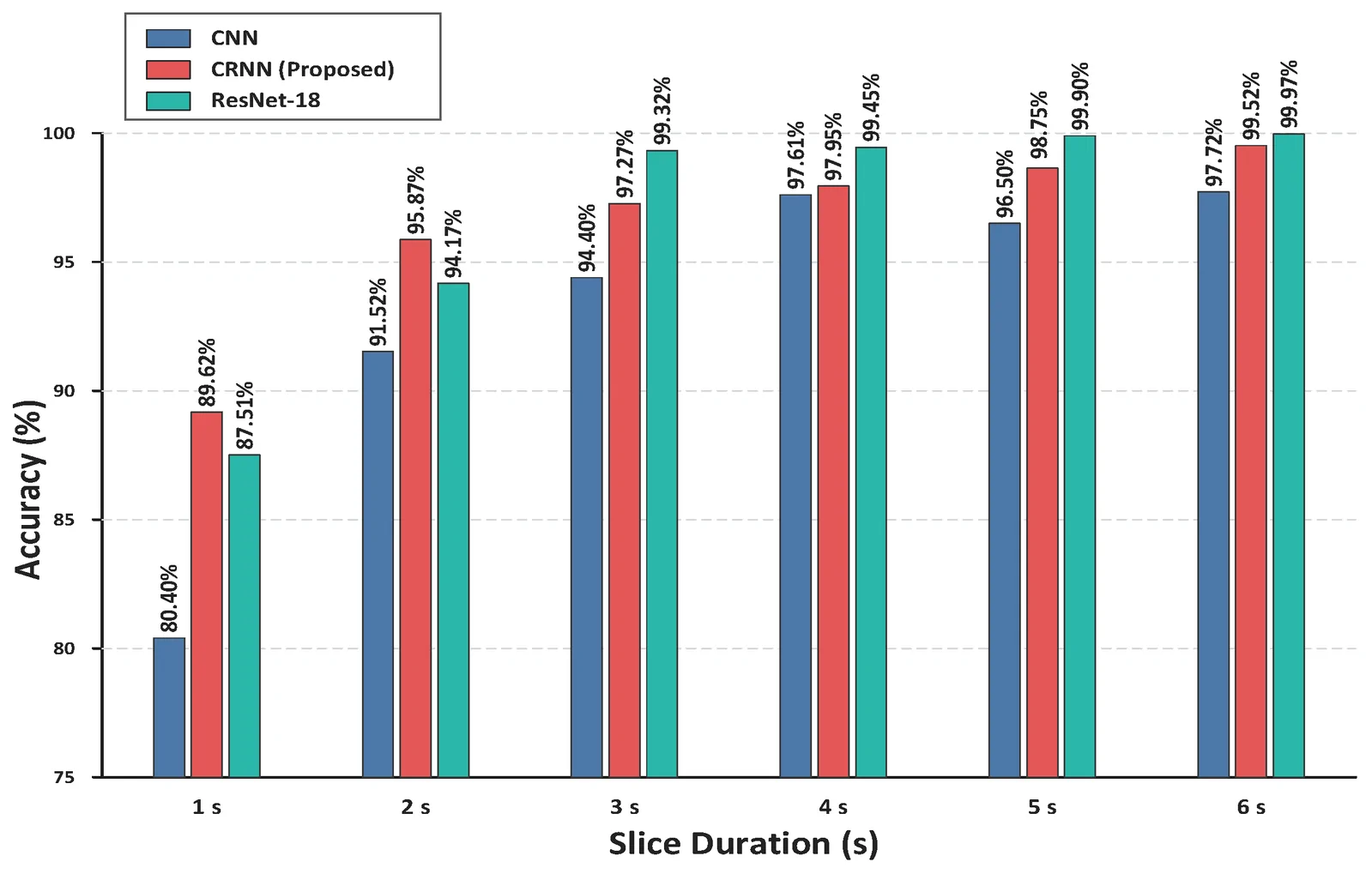
Gait recognition accuracy of various network architectures under different time slice lengths.

**Figure 10:**
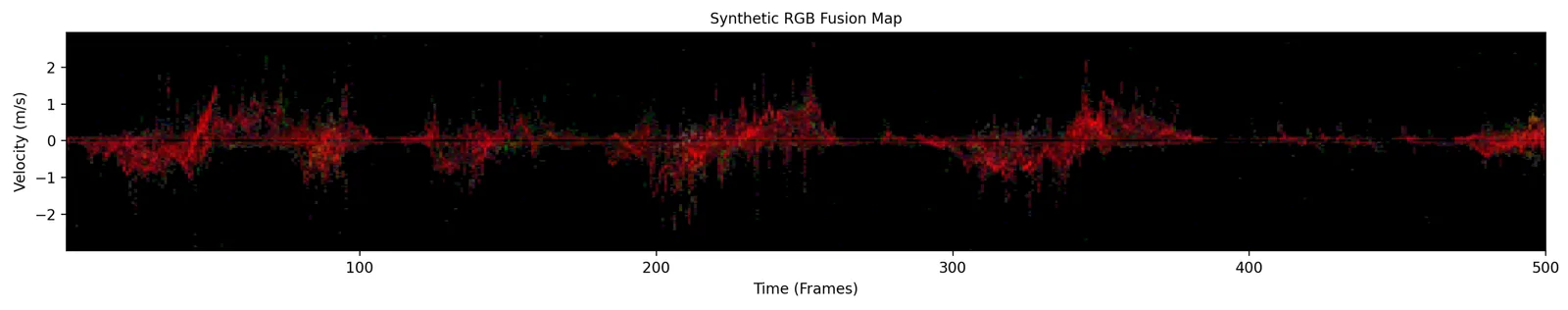
Typical radar micro-Doppler time-frequency spectrogram generated by a pet cat walking freely.

**Figure 11:**
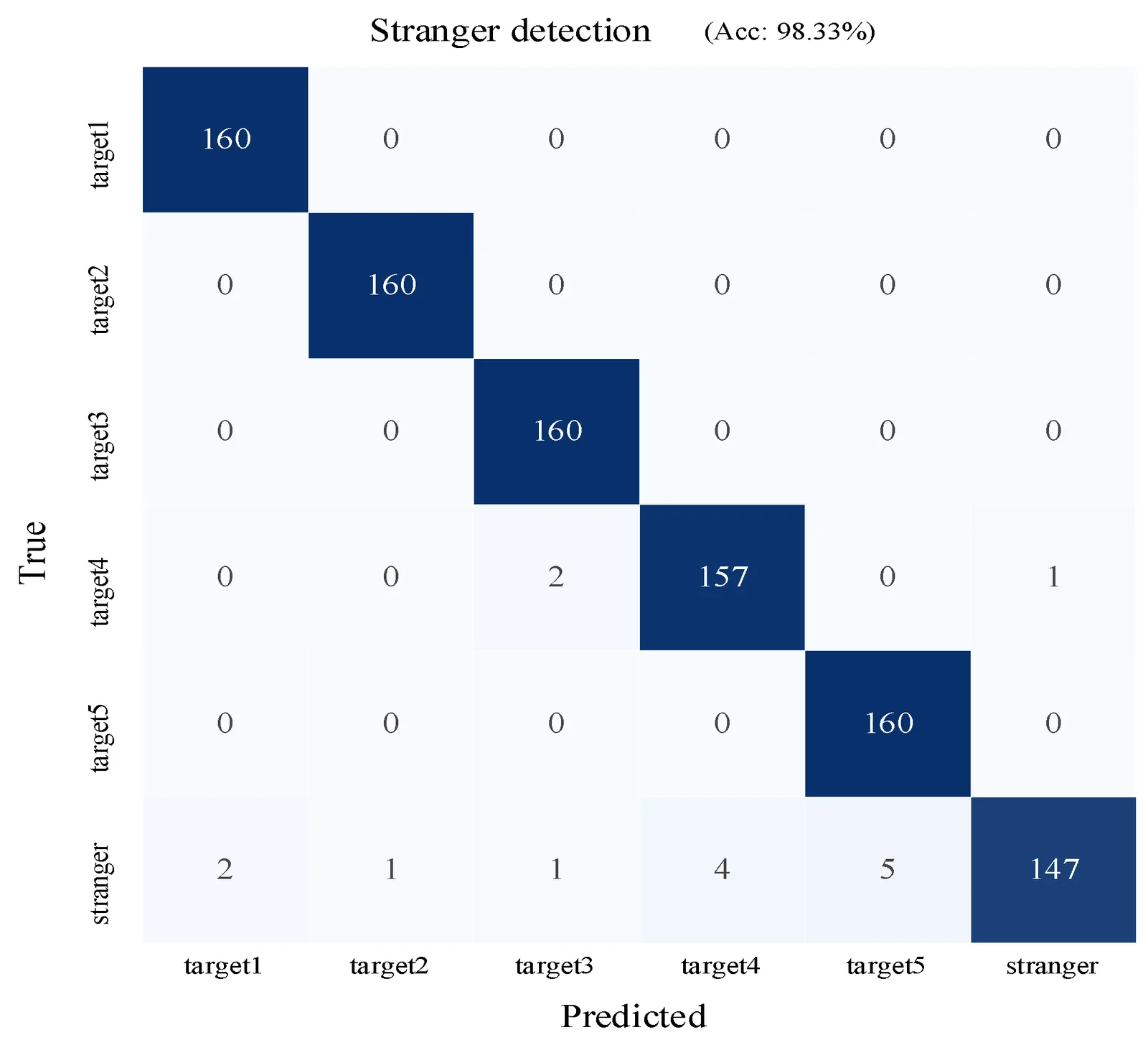
Multi-class confusion matrix in open-set scenarios, covering resident classification and stranger rejection.

**Table 1:** Main acquisition parameters of the TI IWR6843AOP.

Parameter	Value
Start frequency	60 GHz
Frequency slope	99.975 MHz/μs
Operating frequency range	60–64 GHz
Configured sweep bandwidth	4.0 GHz
ADC sampling points	256
Sampling rate	8000 ksps
Frame period	40 ms
TDM loops per frame	128
Idle time	60 μs
Ramp end time	40 μs
ADC start time	6 μs
Chirps per TDM loop	3
Tx activation order	Tx1-Tx2-Tx3
Antenna field of view	120° in azimuth and 120° in elevation
Radar mounting height	2.15 m
Radar orientation	Downward tilt of approximately 15°
Subject-to-radar distance	0.8–6 m

**Table 2:** Ablation results of adaptive Gamma correction and multi-transmit-channel differential encoding using a fixed Asym-CRNN backbone.

Adaptive Gamma	Channel Differential Encoding	1 s Accuracy (%)	6 s Accuracy (%)
×	×	85.58 ± 1.96	91.03 ± 0.66
×	√	86.83 ± 1.71	95.45 ± 2.06
√	×	86.67 ± 1.39	96.47 ± 1.63
√	√	89.62 ± 1.24	99.52 ± 0.80

**Table 3:** Ablation results of different transmit-channel input encodings.

Input Encoding	1 s Accuracy (%)	2 s Accuracy (%)	5 s Accuracy (%)	6 s Accuracy (%)
Tx1 only	86.67 ± 1.39	91.88 ± 0.71	95.92 ± 1.43	96.47 ± 1.63
Direct Tx stacking	87.55 ± 0.45	92.72 ± 1.90	96.80 ± 1.36	98.32 ± 1.09
Signed differential encoding	86.90 ± 1.68	94.25 ± 1.86	96.05 ± 1.77	97.47 ± 0.89
Absolute-difference encoding	89.62 ± 1.24	95.87 ± 1.07	98.75 ± 1.45	99.52 ± 0.80

**Table 4:** Comparison of classification accuracy across different network architectures.

Network Topology Configuration	1 s Accuracy (%)
Sym-CNN	83.62 ± 0.82
Sym-CRNN	85.50 ± 1.76
Asym-CNN	86.75 ± 1.51
Asym-CRNN	89.62 ± 1.24

**Table 5:** System recognition accuracy under short and long observation windows with different numbers of registered subjects.

Number of Registered Subjects	1 s Accuracy (%)	6 s Accuracy (%)
2	98.12 ± 0.14	99.94 ± 0.14
3	95.62 ± 1.72	99.69 ± 1.55
4	93.91 ± 2.10	99.58 ± 1.33
5	89.62 ± 1.24	99.52 ± 0.80

**Table 6:** Experimental settings and performance of representative radar-based gait identification methods for small cohorts.

Methods	Input	Number of Subjects	Observation Window (s)	Evaluation Setting	Performance
Vandersmissen et al. [8]	Micro-Doppler spectrogram	5	3	IDRad; closed set	78.46%
Yoon et al. [4]	Micro-Doppler spectrogram	5	10	IDRad; closed set	91.35%
Feng et al. [10]	Micro-Doppler spectrogram + optoelectronic reservoir computing	5	6.5	IDRad; closed set	84.67%
Dang et al. [5]	Sparse 5D point cloud	9	-	Self-collected; single-person closed set	96.75%
This work	IDRad micro-Doppler	5	3	IDRad; closed set	86.98%
	Self-collected micro-Doppler spectrograms	5 registered, 3 unregistered	6	Self-collected; closed set and open-set rejection	Closed-set accuracy: 99.52%; unknown-person rejection: 91.88%

**Table 7:** Per-window processing time of the Raspberry Pi 5 pipeline (using only 2 cores).

Observation Window (s)	Preprocessing Latency (ms)	Network Inference Latency (ms)	Total Processing Latency (ms)
1	709	6.7	715.7
6	4351	15.2	4366.2

## Data Availability

Due to participant privacy considerations, the radar gait data collected by the institute is not publicly available. Researchers may submit reasonable requests to obtain de-identified data.
